# Quantitative fetal magnetic resonance imaging assessment of cystic posterior fossa malformations

**DOI:** 10.1002/uog.21890

**Published:** 2020-07-01

**Authors:** G. O. Dovjak, M. C. Diogo, P. C. Brugger, G. M. Gruber, M. Weber, S. Glatter, R. Seidl, D. Bettelheim, D. Prayer, G. J. Kasprian

**Affiliations:** ^1^ Department of Biomedical Imaging and Image‐Guided Therapy Medical University of Vienna Vienna Austria; ^2^ Department of Anatomy and Biomechanics Karl Landsteiner University of Health Sciences Krems Austria; ^3^ Department of Pediatrics and Adolescent Medicine Medical University of Vienna Vienna Austria; ^4^ Department of Obstetrics and Feto‐Maternal Medicine Medical University of Vienna Vienna Austria

**Keywords:** brain segmentation, cerebellar dysplasia, cerebellar vermis, fetal MRI, hindbrain malformation, MRI, neurodevelopment, vermian lobulation

## Abstract

**Objective:**

Normal cognitive development usually requires a structurally intact and complete cerebellar vermis. The aim of this study was to evaluate whether quantification by fetal magnetic resonance imaging (MRI) of vermis‐ and brainstem‐specific imaging markers improves the definition of cystic posterior fossa malformations (cPFM).

**Methods:**

Fetuses diagnosed with cPFM that had an available midsagittal plane on T2‐weighted MRI were identified retrospectively and compared with gestational‐age (GA) matched brain‐normal controls. Fetuses with cPFM were assigned to three groups, according to standard criteria (vermian size and brainstem–vermis (BV) angle): normal vermian area and BV angle < 25**°** (Group 1); reduced vermian area and/or BV angle of 25–45° (Group 2); and reduced vermian area and BV angle > 45° (Group 3; Dandy–Walker malformation (DWM) group). The number of differentiable vermian lobules and the areas of the vermis, mesencephalon, pons and medulla oblongata were quantified, correlated with and controlled for GA, and compared between the study groups.

**Results:**

In total, 142 cases of cPFM were included, with a mean GA of 25.20 ± 5.11 weeks. Cases comprised Blake's pouch cyst (*n* = 46), arachnoid cyst (*n* = 12), inferior vermian hypoplasia (*n* = 5), megacisterna magna (*n* = 35) and classic DWM (*n* = 44). In the control group, 148 fetuses were included, with a mean GA of 25.26 ± 4.12 weeks. All quantified areas and the number of differentiable vermian lobules had a significant positive correlation with GA. The number of vermian lobules and the areas of all quantified regions, except for that of the medulla oblongata, differed significantly between the study groups (*P* ≤ 0.015 for all). The control group had the highest number of differentiable vermian lobules and the DWM group had the lowest (*P* < 0.01).

**Conclusions:**

Prenatal MRI assessment of vermian lobules is a useful addition to standard neuroradiological and neurosonographic techniques. The quantification of vermian lobules using fetal MRI allows further differentiation of cPFM into subgroups and thereby improves the classification of hindbrain malformations. © 2019 The Authors. *Ultrasound in Obstetrics & Gynecology* published by John Wiley & Sons Ltd on behalf of the International Society of Ultrasound in Obstetrics and Gynecology.

**CONTRIBUTION**

*What are the novel findings of this work?*

This fetal magnetic resonance imaging (MRI) study quantified posterior fossa structures in fetuses with cystic posterior fossa malformations and correlated them with gestational age and the current standard pathological classification. Using this quantification, particularly with respect to vermian lobulation, different subgroups of cystic posterior fossa malformation can be differentiated.

*What are the clinical implications of this work?*

Prenatal MRI assessment of vermian lobules is a useful addition to standard neuroradiological and neurosonographic parameters. Quantification of vermian lobules using fetal MRI allows further differentiation of cystic posterior fossa malformations into subgroups and thereby improves the classification of hindbrain malformations. This should have an impact on neurodevelopmental outcome and parental counseling.

## INTRODUCTION

The cerebellum contains numerous afferences and efferences involved in different functional systems, including affective, cognitive and motor processing^1^, ^2^. Hindbrain malformations comprise a group of genetically and developmentally diverse disorders^3^, ^4^, ^5^, the clinical manifestations of which range from normal neurocognitive development to severe psychomotor delay^6^, ^7^. Wide variations and misconceptions in the nomenclature of cystic posterior fossa malformations (cPFM) further add to the difficulty of obtaining a precise, prognostically valuable prenatal diagnosis^8^, ^9^, and classification, outcome prediction and prenatal counseling are generally difficult in these situations. Assessment of cPFM requires standardized categorization and a multidisciplinary approach^10^.

The present classification of cPFM is based on morphologic criteria (position of torcula, associated brain malformations) and quantitative evaluation (vermian area, transcerebellar diameter, brainstem–vermis (BV) angle). cPFM are usually identified by prenatal ultrasound screening, however, a definitive diagnosis of a malformation may be difficult. Owing to the absence of acoustic shadowing, fetal magnetic resonance imaging (MRI) allows consistent and dedicated assessment of the cerebellum and even its small structures, such as the vermian lobules^11^. The morphology of the cerebellar vermis and in particular the number of vermian lobules have been linked to neurodevelopmental outcome^12^.

Barkovich *et al*.^13^ classified cPFM as ‘mesenchymal–neuroepithelial signaling defects associated with mid‐hindbrain malformations’. Prenatal ultrasound is able to recognize only five conditions within that entity^14^: megacisterna magna (MM), Dandy–Walker malformation (DWM), arachnoid cysts (AC), vermian hypoplasia (VH) and Blake's pouch cyst (BPC)^15^. A more morphologically detailed analysis of cPFM could open up possibilities for their further characterization and for optimizing their prenatal classification.

To improve the characterization of cPFM, this retrospective fetal MRI study assessed a variety of imaging markers in the posterior fossa of fetuses with a prenatal ultrasound diagnosis of cPFM. The main aim was to determine whether further prenatal differentiation of subgroups of cPFM is possible based on the number of visible vermian lobules on MRI. The main hypothesis was that assessment of vermian lobules would allow further differentiation of the three main prognostic groups, as defined by currently used standard quantitative parameters.

## METHODS

This retrospective study was approved by the local institutional review board of the Medical University of Vienna (ethics committee number 2122/2017). All examined subjects had given written, informed consent for the scientific use of their anonymized imaging data.

### Study subjects

Potential fetal subjects with cPFM and an exact, midsagittal slice on a T2‐weighted MRI sequence, performed between May 2003 and May 2018, were identified retrospectively in the hospital's database. The MRI plane was defined by an image that depicted the corpus callosum, brainstem and cerebellar vermis in their entirety. All cases were referred for fetal MRI after a standard neurosonographic examination by an obstetrician who was a level II or III sonographer by European standards. Subjects with marked motion artifacts and multiple pregnancies were excluded.

As a reference standard, age‐matched fetuses with normal structural brain development on ultrasound and MRI from an existing database were included^11^. Indications for MRI in this group were mainly body malformations and premature rupture of membranes.

### Magnetic resonance imaging technique

Fetal MRI was performed using a 1.5‐T (Ingenia; Philips Medical Systems) or 3‐T (Achieva; Philips Medical Systems, Best, The Netherlands) scanner. A body coil was used and exact, midsagittal T2‐weighted turbo‐spin echo images were obtained with the following specifications: acquired in‐plane resolution ranging from 0.62/0.62 mm to 1/1 mm with a slice thickness of 2.0 to 4.4 mm, matrix of 256 × 256, field of view of 200–230 mm, relaxation time of ≤ 20 000 ms and echo time of 100–140 ms.

### Image evaluation

Fetuses with a cPFM were assigned to one of the three following groups, according to the criteria of Gandolfi Colleoni *et al*.^14^, depending on BV angle and vermian size^16^: normal vermian area and BV angle < 25° (Group 1); reduced vermian area and/or BV angle of 25–45° (Group 2); and reduced vermian area and BV angle > 45° (Group 3; DWM group).

**Figure 1:**
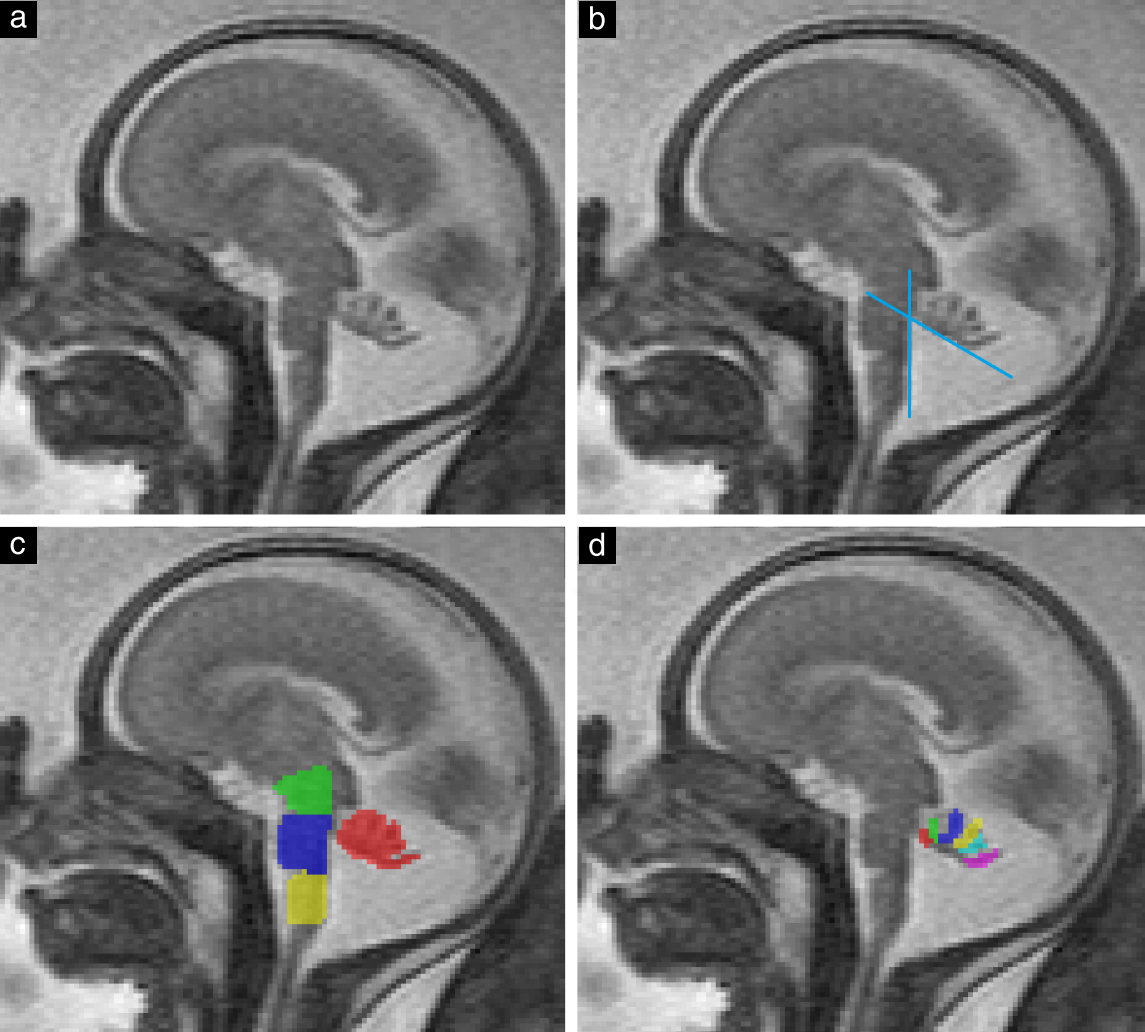
(a) Midsagittal T2‐weighted magnetic resonance image of fetal brain at 23 + 4 weeks' gestation. (b) Brainstem–vermis angle (here, 60°) measured between dorsal border of brainstem at level of pons and ventral tangent of uprotated cerebellar vermis. (c) Areas of vermis (red), mesencephalon (green), pons (blue) and medulla oblongata (yellow) were segmented. (d) Number of differentiable lobules (six in this case) was assessed.

One observer (G.D.), with 3 years' experience of fetal MRI, evaluated all subjects in both groups. The following parameters were assessed quantitatively using the open‐source software application ITK‐SNAP (www.itksnap.org), version 3.6^17^ (Figure 1): number of differentiable vermian lobules, BV angle, midsagittal vermian area, area of brainstem structures (mesencephalon, pons and medulla oblongata), presence of vermian tail sign, presence of external mechanical pressure on the vermis, differentiability of the declive, folium and tuber (DFT) and differentiability of the uvula from the nodulus. The vermian tail sign^18^ was described initially in DWM and could further help in distinguishing it from other cPFM that show gross anatomic similarities (for example, if the vermis is uprotated by Blake's pouch, it can be confused with DWM^19^).

**Figure 2:**
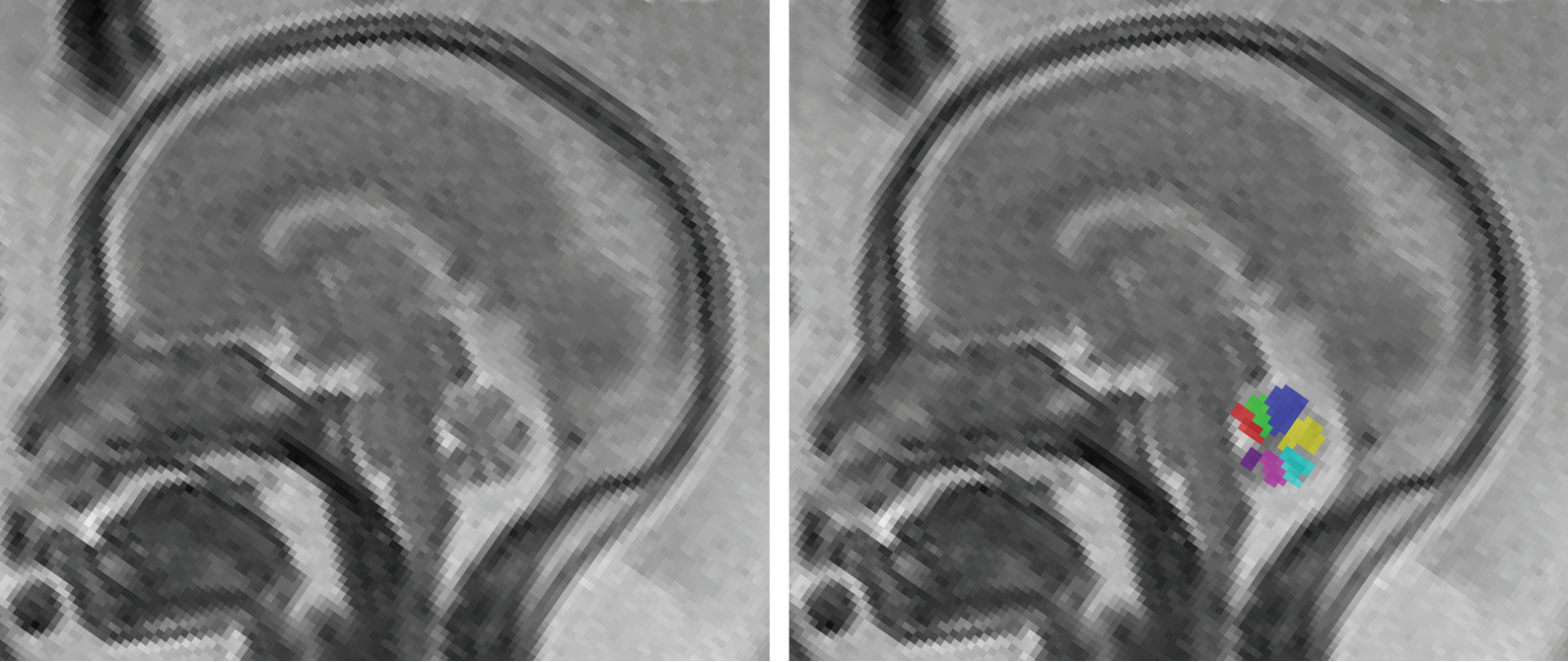
Midsagittal T2‐weighted magnetic resonance image of normal fetal brain at 22 + 0 weeks' gestation, showing seven (of nine anatomically possible) differentiable vermian lobules segmented (colored areas). In older fetuses, up to nine lobules can be differentiated.

In this study, BV angle was measured between the posterior border of the brainstem at the level of the pons and the tangent of the ventral vermis (Figure 1). The number of vermian lobules was assessed in accordance with a previous study in brain‐normal controls^11^. For the definition of cerebellar lobules and fissures, the nomenclature proposed by Schmahmann *et al*.^20^ was used as an anatomical reference. The cerebellar vermis consists of nine separable lobules^21^, but in the present study the maximum number of differentiable lobules was taken to be seven (Figure 2), because the three lobules of the posterior lobe (the DFT) could not be differentiated in any brain‐normal fetal cases in a prior study^11^. We determined in how many cases the DFT could be differentiated into two or three lobules. Whether the uvula and nodulus were differentiable was also assessed, as these lobules are difficult to differentiate in some cases owing to volume averaging with the cerebellar tonsil from the hemispheres.

Segmentation of brainstem areas (Figure 1c) was performed as proposed by Oba *et al*.^22^. The pons was segmented between a rostral line from the pontomesencephalic junction (the ‘superior pontine notch’) to the inferior colliculus of the quadrigeminal plate, and a caudal line perpendicular to the bulbopontine sulcus. The mesencephalon was segmented between a rostral line from the cranial part of the superior colliculus to the mamillary body and the pons, excluding the quadrigeminal plate. The tectum was excluded from segmentation because of its inconsistent midline and varying thickness. The medulla oblongata was segmented between the pons and the obex.

Two additional observers (G.K. and M.D.), with 15 and 6 years of experience with fetal MRI, respectively, independently evaluated the number of vermian lobules in the pathological groups in order to assess inter‐rater differences. The two additional observers also independently evaluated the presence of mechanical pressure on the vermis. In all analyses, the number of lobules in fetuses in the pathological groups represents the mean value recorded by the three observers.

### Statistical analysis

Statistical analysis was carried out using SPSS Statistics for Windows, v. 25 (IBM Corp., Armonk, NY, USA). A sample size calculation using NQuery Advanced (www.statsols.com/nquery) revealed that a total sample size of 152 cases (expected distribution of the three groups of 3:2:1) would be needed to obtain a power of 80% to detect the expected effect size of delta^2^ = 0.065.

Nominal data were described as absolute frequencies and percentages. Mean and SD were calculated for gestational age (GA) in weeks. Pearson's correlation coefficient was used to assess the correlation between GA, number of vermian lobules and all quantified areas. Analysis of covariance (ANCOVA) was used to compare the areas of the quantified regions and the number of differentiable vermian lobules between the groups, corrected for GA, and estimated marginal means with SEs were used as descriptive statistics. Multinomial logistic regression analysis was used to determine which variables had a significant impact on the correct classification of the groups.

To assess for differences in area measurements and the number of vermian lobules between fetuses that had 1.5‐T MRI and those that had 3‐T MRI, a paired *t*‐test based on age‐matched pairs was performed within the relatively homogeneous control group.

The intraclass correlation coefficient (ICC) was calculated for assessment of the number of vermian lobules by the three observers in the pathological groups in order to test inter‐rater variability and reliability; an ICC above 0.75 was considered to indicate excellent correlation^23^. *P* ≤ 0.05 was considered to indicate a statistically significant result. To avoid increasing the risk of type‐2 error, multiplicity corrections were used for *post‐hoc* tests only.

## RESULTS

**Figure 3:**
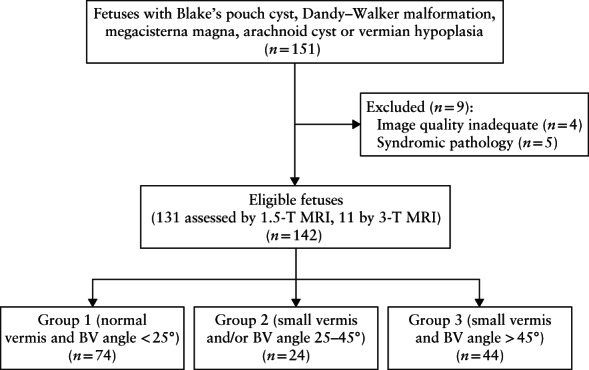
Flowchart summarizing inclusion of fetuses with cystic posterior fossa malformation. BV, brainstem–vermis.

In the brain‐normal control group, 148 fetuses were included; GA ranged from 18 to 40 weeks, with a mean ± SD of 25.26 ± 4.12 weeks. In the pathological group, 151 fetuses (Figure 3) were identified after screening existing fetal MRI reports for cases with a diagnosis of BPC, DWM, MM, AC or VH. Five syndromic/complex cases were excluded and four cases were omitted owing to inadequate image quality (oblique image, no available midsagittal image, motion blurring), resulting in a total of 142 eligible fetuses, with a GA of 16–40 weeks and a mean of 25.20 ± 5.11 weeks. Based on the existing morphologic criteria, 46 fetuses were diagnosed with BPC, 44 with DWM, 35 with MM, 12 with AC and five with VH.

Group 1 (*n* = 74), defined as having a BV angle of < 25° and normal vermian morphology, had a GA of 26.87 ± 4.78 weeks and comprised cases of MM, AC and BPC. Group 2 (*n* = 24), defined as having a small vermis and/or increased BV angle of 25–45°, had a GA of 23.46 ± 5.45 weeks and consisted of cases of VH and BPC. The DWM group (Group 3) (*n* = 44), defined as having increased BV angle of > 45° and reduced vermian area, had a GA of 23.42 ± 4.49 and included cases of DWM only.

Table 1 shows the groupwise distribution of the number of vermian lobules when categorized into three groups: 1–3 lobules, 4–5 lobules or 6–7 lobules. In the control group, 87.8% (130/148) showed six or more differentiable lobules. In Group 1, about two‐thirds (62.2% (46/74)) showed six or more differentiable lobules. More than half the fetuses in Group 2 (58.3% (14/24)) had four or five lobules. The distribution of lobules in the DWM group was bell shaped, as nearly half the fetuses (45.5% (20/44)) had four or five differentiable lobules, 25.0% (11/44) had six or more and 29.5% (13/44) had three or fewer.

**Table 1:** Distribution of number of vermian lobules on magnetic resonance imaging in fetuses with cystic posterior fossa malformations, according to group defined by standard criteria (vermian size and brainstem–vermis (BV) angle), and in brain‐normal controls

Number of lobules	Controls (*n* = 148)	Group 1 (*n* = 74)	Group 2 (*n* = 24)	Group 3 (*n* = 44)
1–3	0 (0.0)	0 (0.0)	0 (0.0)	13 (29.5)
4–5	18 (12.2)	28 (37.8)	14 (58.3)	20 (45.5)
6–7	130 (87.8)	46 (62.2)	10 (41.7)	11 (25.0)

Data are given as *n* (%).

In Groups 1–3, number of lobules is based on mean value of three observers.

Groups defined as follows: normal vermian area and BV angle < 25° (Group 1); reduced vermian area and/or BV angle of 25–45° (Group 2); reduced vermian area and BV angle > 45° (Group 3; Dandy–Walker malformation group).

All quantified areas and the number of differentiable vermian lobules had a significant positive correlation with GA, Pearson's correlation coefficient ranging from 0.78 to 0.97 (*P* < 0.01 for all). The number of vermian lobules showed a significant correlation with each of the evaluated areas in all groups, with correlation coefficients ranging from 0.44 to 0.72 (*P* < 0.01 for all), except for the DWM group, in which only vermian area was correlated significantly with the number of lobules (Table 2).

**Table 2:** Correlation between areas of vermis and brainstem regions evaluated quantitatively on magnetic resonance imaging and number of differentiable vermian lobules in fetuses with cystic posterior fossa malformations, according to group defined by standard criteria (vermian size and brainstem–vermis (BV) angle), and in brain‐normal controls

Region	Controls (*n* = 148)		Group 1 (*n* = 74)		Group 2 (*n* = 24)		Group 3 (*n* = 44)	
	*r*	*P*	*r*	*P*	*r*	*P*	*r*	*P*
Vermis	0.596	< 0.001	0.499	< 0.001	0.669	< 0.001	0.464	0.002
Mesencephalon	0.623	< 0.001	0.437	< 0.001	0.524	0.009	0.164	0.288
Pons	0.614	< 0.001	0.508	< 0.001	0.716	< 0.001	0.223	0.146
Medulla oblongata	0.635	< 0.001	0.479	< 0.001	0.615	0.001	0.226	0.141

Groups defined as follows: normal vermian area and BV angle < 25° (Group 1); reduced vermian area and/or BV angle of 25–45° (Group 2); reduced vermian area and BV angle > 45° (Group 3; Dandy–Walker malformation group).

*r*, Pearson's correlation coefficient.

**Figure 4:**
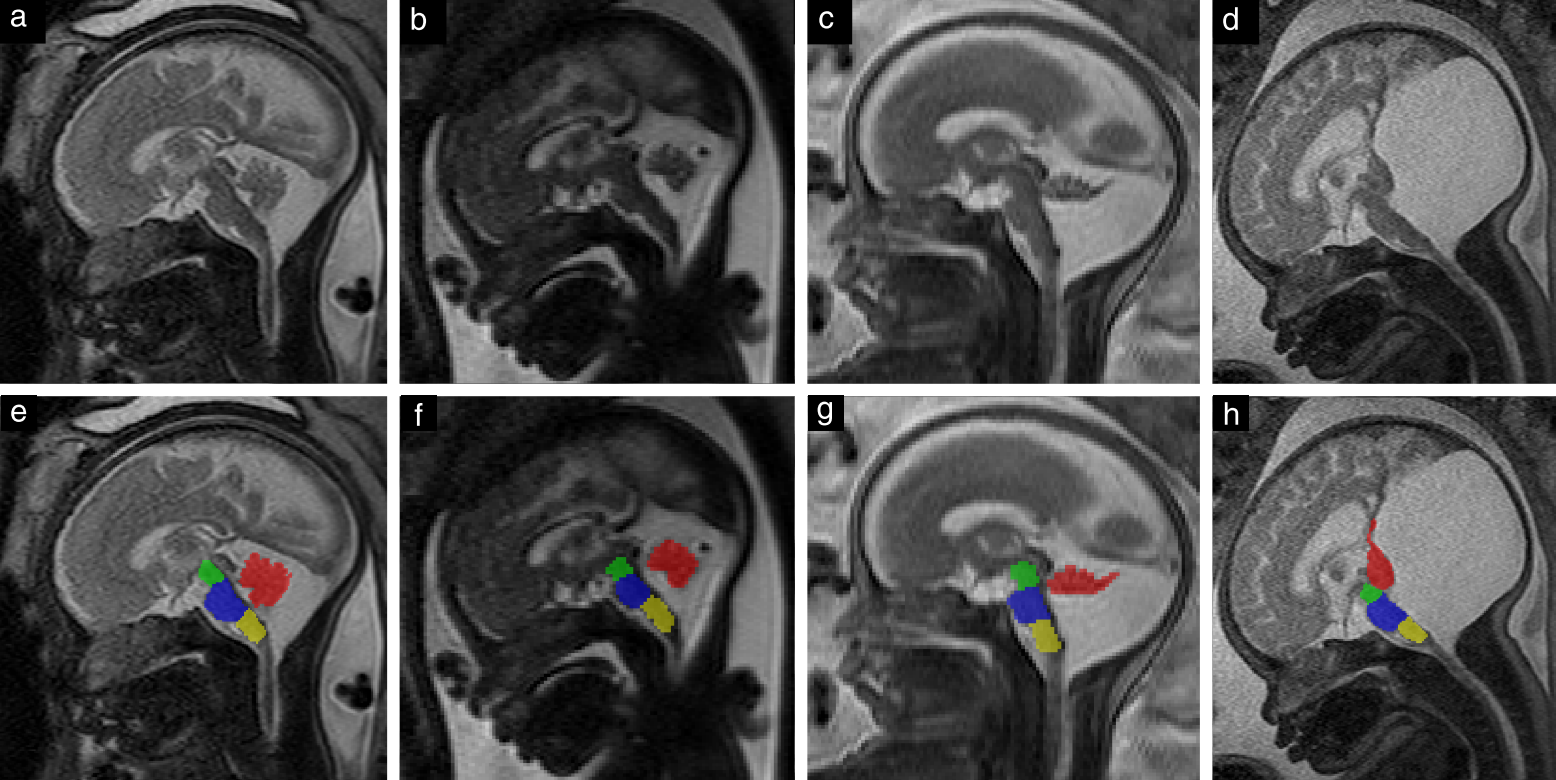
Midsagittal T2‐weighted magnetic resonance images in fetuses with cystic posterior fossa malformation (a–d), and with corresponding segmentation of vermis (red), mesencephalon (green), pons (blue) and medulla oblongata (yellow) (e–h). (a,e) Group‐1 fetus at 32 + 4 weeks' gestation; (b,f) Group‐2 fetus at 29 + 2 weeks; (c,g) Group‐3 (Dandy–Walker malformation group) fetus at 25 + 4 weeks; (d,h) Group‐3 fetus (with more distinct findings) at 36 + 3 weeks.

Table 3 shows the mean values of the assessed parameters in each group, after controlling for the effect of GA using analysis of covariance. There was a significant difference between groups in the number of vermian lobules and the area of the vermis, mesencephalon and pons (*P* < 0.001 to 0.015). The area of the medulla oblongata did not show a significant difference between groups (*P* = 0.236). The number of vermian lobules, as well as the area of the vermis, decreased across the groups, with the control group having the highest number of lobules and the largest area, and the DWM group having the lowest. The area of the mesencephalon and pons had the same pattern but with the last two groups switched (Group 2 had the smallest values and the DWM group had the second smallest). Figure 4 shows MRI scans of fetal brains in all groups, with segmentation of the quantified areas.

**Table 3:** Estimated marginal means of vermis‐ and brainstem‐specific markers on magnetic resonance imaging in fetuses with cystic posterior fossa malformations, according to group defined by standard criteria (vermian size and brainstem–vermis (BV) angle), and in brain‐normal controls

Variable	Controls (*n* = 148)	Group 1 (*n* = 74)	Group 2 (*n* = 24)	Group 3 (*n* = 44)	*P*
Number of vermian lobules*	6.52 ± 2.68	5.61 ± 2.68	5.05 ± 2.68	4.11 ± 2.68	< 0.001
Area of vermis (mm^2^)	118.09 ± 17.11	110.05 ± 17.20	93.78 ± 17.53	81.40 ± 17.31	< 0.001
Area of mesencephalon (mm^2^)	41.16 ± 0.65	40.05 ± 0.96	36.16 ± 1.63	38.18 ± 1.23	0.015
Area of pons (mm^2^)	77.41 ± 9.56	73.36 ± 9.62	67.74 ± 9.85	72.33 ± 9.70	0.001
Area of medulla oblongata (mm^2^)	48.95 ± 10.15	49.01 ± 10.18	48.10 ± 10.29	45.90 ± 10.22	0.236

Data are given as mean ± SD for average gestational age of 25.23 weeks.

Effect of gestational age on parameters was eliminated using analysis of covariance.

* In Groups 1–3, number of lobules is based on mean value of three observers.

Groups defined as follows: normal vermian area and BV angle < 25° (Group 1); reduced vermian area and/or BV angle of 25–45° (Group 2); reduced vermian area and BV angle > 45° (Group 3; Dandy–Walker malformation group).

Table S1 shows *P*‐values of pairwise comparisons between groups for each quantitative parameter, after elimination of the effect of GA on the data. The control group had significantly more lobules than did all other groups (*P* < 0.001), and Group 3 had significantly fewer lobules than did all other groups (*P* < 0.001). Group 1 had more lobules than did Group 2 but the difference was not significant (*P* = 0.053). Vermian area decreased significantly from the control group to Group 2 (*P* < 0.001 to 0.041). Group 2 had a non‐significantly larger vermian area than did the DWM group (*P* = 0.102). The areas of the mesencephalon and pons were significantly larger in the control group than in Group 2 (*P* = 0.029 and 0.004, respectively). There was no significant difference in the area of the medulla oblongata between any of the groups.

All evaluated binary variables are listed in Table 4. DFT could be distinguished in 66.9% of controls, 32.4% of cases in Group 1, 8.3% of those in Group 2 and in none of those in the DWM group. Analogously, the uvula and nodulus were distinguishable in 87.8% of controls, 37.8% of cases in Group 1, 16.7% of those in Group 2 and 13.6% of those in the DWM group. The vermian tail sign was not seen in any of the controls, but it was visible in 1.4% of cases in Group 1, 45.8% of those in Group 2 and 75.0% of those in the DWM group. There was evidence of mechanical pressure on the vermis in 36.4% of cases in the DWM group and in 5.4% of those in Group 1.

**Table 4:** Frequency of binary vermis‐ and brainstem‐specific imaging markers on magnetic resonance imaging in fetuses with cystic posterior fossa malformations, according to group defined by standard criteria (vermian size and brainstem–vermis (BV) angle), and in brain‐normal controls

Variable	Controls (*n* = 148)	Group 1 (*n* = 74)	Group 2 (*n* = 24)	Group 3 (*n* = 44)
Declive, folium and tuber distinguishable	99 (66.9)	24 (32.4)	2 (8.3)	0 (0.0)
Uvula and nodulus distinguishable	130 (87.8)	28 (37.8)	4 (16.7)	6 (13.6)
Vermian tail sign	0 (0.0)	1 (1.4)	11 (45.8)	33 (75.0)
Mechanical pressure on vermis	0 (0.0)	4 (5.4)	0 (0.0)	16 (36.4)

Data are given as *n* (%).

Groups defined as follows: normal vermian area and BV angle < 25° (Group 1); reduced vermian area and/or BV angle of 25–45° (Group 2); reduced vermian area and BV angle > 45° (Group 3; Dandy–Walker malformation group).

In the control group, 139 (93.9%) fetuses were examined using a 1.5‐T MRI scanner and nine (6.1%) using a 3‐T scanner. A paired, age‐matched *t*‐test was possible for seven pairs of controls with similar ages. No significant difference in the number of vermian lobules, vermian area or areas of the brainstem substructures was seen when using the two different field strengths. The fetuses in the pathological group were distributed similarly, as 131 (92.3%) fetuses had 1.5‐T MRI and 11 (7.7%) fetuses had 3‐T MRI.

The ICC for assessment of the number of vermian lobules between the first and second observers was 0.892, between the first and third observers it was 0.836 and between the second and third observers it was 0.778, which was considered excellent correlation.

Multinomial logistic regression analysis showed that the groups were assigned correctly in 62.2% of cases when based solely on vermian area, and in 68.5% when based on the number of vermian lobules only.

After exclusion of all cases with additional body and brain malformations (Table S2), the number of vermian lobules still differed between groups. There was no significant difference in the number of lobules between fetuses with and those without additional findings (*P* = 0.616).

## DISCUSSION

Recent advances in fetal imaging and, specifically, fetal MRI, allow for accurate and reliable delineation of the vermian lobules^11^. Characterization and quantification of vermian structures are relevant for the diagnosis of morphologically different subgroups of cPFM, as these subgroups may explain the well‐known heterogeneity in the neurodevelopmental outcomes of affected cases. The findings of this retrospective fetal MRI study, in which a variety of posterior fossa structures were assessed quantitatively, show that detailed analysis of the fetal vermis is possible and allows further classification of cPFM into subgroups.

The number of vermian lobules in the existing standardized diagnostic groups was clustered, as most fetuses in the brain‐normal group and Group 1 (comprising MM, AC and BPC) had six or more differentiable lobules and most fetuses in Group 2 (comprising VH and BPC) had four or five lobules. On the other hand, the DWM group showed a scattered distribution pattern, as most (45.5%) fetuses had four or five differentiable lobules, 11 (25.0%) had more and 13 (29.5%) had fewer.

The pathologies were assigned to three previously well defined groups following standard quantitative prenatal biometry markers (BV angle and vermian size)^4^. The size of the BV angle is thought to be an important distinguishing marker, with DWM showing more prominent rotation than BPC^24^. Evaluation of 142 fetuses in the pathological groups showed that the number of vermian lobules and vermian area differed significantly between the previously defined groups. Moreover, the differences in these parameters became further evident as they were found to be heterogeneous within certain groups.

The excellent inter‐rater agreement (ICC ≥ 0.778) for assessment of the number of vermian lobules in the pathological groups proves that quantification of vermian lobules using fetal MRI is possible and reliable not only in brain‐normal fetuses, but also in cases of hindbrain malformations.

As Klein *et al*.^12^ showed that children with DWM with more differentiable lobules had a better neurodevelopmental outcome, our findings, if confirmed by dedicated neuropediatric follow‐up, are promising morphological markers and may have a significant impact on prenatal counseling. No fetus in the control group, Group 1 or Group 2 had fewer than four differentiable vermian lobules, making a cut‐off of three or fewer lobules a possible indicator of rather ‘worse’ postnatal development.

Within the DFT lobule, which is known to be correlated with cognitive memory^25^, ^26^, the individual lobules (declive, folium and tuber) could be differentiated in two‐thirds of controls and in one‐third of cases in Group 1.

The number of vermian lobules correlates significantly with the vermian area and the area of brainstem substructures, indicating that, with advancing gestation, more lobules can be differentiated owing to increasing vermian size. It has been shown that seven lobules can be detected reliably after 22 weeks' gestation in brain‐normal fetuses^11^, also indicating that the number of differentiable lobules depends on vermian size. In the DWM group, the number of vermian lobules was correlated with vermian area only. This can be attributed to the fact that, in non‐syndromic DWM, there is a discrepancy between a moderate to severe dysplastic vermis in contrast to a normally configured to moderately hypoplastic brainstem^27^. Consequently, the lobule number correlated only with the hypoplastic vermis.

The vermian tail sign, which has been reported previously as a potential parameter for distinguishing DWM from other cPFM^18^, was non‐specific to the DWM group, as 45.8% of Group 2 (comprising VH and BPC) and 1.4% of Group 1 (comprising MM, AC and BPC) also demonstrated this sign. Mechanical pressure on the vermis, which narrows the distance between lobules and leads to a lower number of differentiable lobules, was present mainly in the DWM group (36.4%), but also in a few cases (5.4%) in Group 1. The amount of mechanical pressure could thus have an impact on the lobule count and should be included in the evaluation of cPFM.

We did not find any significant differences in measurements between control fetuses assessed using a 1.5‐T MRI scanner and those assessed using a 3‐T scanner, which could be attributable to the low power due to the small number (*n* = 9) of controls that had 3‐T MRI.

An inherently limiting factor of *in‐vivo* fetal cerebellar imaging is the small vermian size, with a craniocaudal diameter of about 1.1 cm at 20 weeks' gestation^28^. The size and limited resolution make segmentation of posterior fossa structures prone to partial volume effects. The area of the medulla oblongata showed a relatively large SD compared with its mean value (Table 3), which can be attributed to its inconsistent height and to measurement errors, as the obex is difficult to identify. Although movement artifacts can be a limiting factor in fetal MRI, we were able to bypass them by repeating the desired image plane. Another potential limitation is the lack of postmortem, postnatal correlation and neurodevelopmental outcome. However, these correlations are of limited value, as the cerebrospinal fluid (CSF) spaces in postmortem fetuses decrease and children often have a ventricular shunt, which also alters the CSF spaces (and the position of the vermis)^29^. The outcome of fetuses was not assessed in this retrospective study and will be a topic for further prospective research correlating vermian morphology (in particular vermian lobulation) with neurocognitive outcome. Systematic long‐ and short‐term postnatal follow‐up evaluation is currently ongoing. However, the current data do not allow for meaningful imaging, clinical and/or postmortem correlation.

In conclusion, fetal MRI‐based assessment of vermian lobules and quantitative evaluation of the brainstem and vermian areas is a useful tool to further differentiate and characterize cPFM. The number of vermian lobules differed significantly between the assessed standard diagnostic groups. This is of importance, as malformations with known worse postnatal outcomes tend to have fewer differentiable vermian lobules^30^. However, follow‐up studies are needed to correlate these findings with postnatal neurodevelopmental outcomes in order to improve parental counseling. In future, a more detailed qualitative analysis of the prenatal appearance of cerebellar lobules in cPFM may lead to the identification of subtle changes that are specific for certain types of hindbrain malformation^31^.

## Supporting information


**Table S1** Pairwise Bonferroni‐corrected *P*‐values for comparisons of vermis‐ and brainstem‐specific markers on magnetic resonance imaging between fetuses with cystic posterior fossa malformations, according to group defined by standard criteria (vermian size and brainstem–vermis (BV) angle), and brain‐normal controls
**Table S2** Number of additional pathologic findings in study groups
