# Supplementary material for: Quantitative fetal magnetic resonance imaging assessment of cystic posterior fossa malformations
Source: Ultrasound Obstet Gynecol. 2020 Jul 1;56(1):78–85. doi: 10.1002/uog.21890 (PMC7384051; doi:10.1002/uog.21890)
Supplement: Supplementary file 1 — Table S1 Pairwise Bonferroni‐corrected P‐values for comparisons of vermis‐ and brainstem‐specific markers on magnetic resonance imaging between fetuses with cystic posterior fossa malformations, according to group defined by standard criteria (vermian size and brainstem–vermis (BV) angle), and brain‐normal controls Table S2 Number of additional pathologic findings in study groups [file UOG-56-78-s001.docx]

**Table S1** Pairwise Bonferroni-corrected P-values for comparisons of vermis- and brainstem-specific markers on magnetic resonance imaging between fetuses with cPFM, according to group defined by standard criteria (vermian size and brainstem-vermis (BV) angle), and brain-normal controls

|  | Controls vs: | | | Group 1 vs: | |  |
| --- | --- | --- | --- | --- | --- | --- |
| Variable | Group 1 | Group 2 | Group 3 | Group 2 | Group 3 | Group 2 vs group 3 |
| Number of vermian lobules | <0.001 | <0.001 | <0.001 | 0.053 | <0.001 | <0.001 |
| Area of vermis (in mm^2^) | 0.041 | <0.001 | <0.001 | 0.006 | <0.001 | 0.102 |
| Area of mesencephalon (in mm^2^) | 1 | 0.029 | 0.201 | 0.257 | 1 | 1 |
| Area of pons (in mm^2^) | 0.17 | 0.004 | 0.131 | 0.386 | 1 | 0.922 |
| Area of medulla oblongata (in mm^2^) | 1 | 1 | 0.288 | 1 | 0.452 | 1 |

Effect of gestational age on parameters was eliminated using analysis of covariance. Groups defined as follows: normal vermian area and BV-angle ≤ 25° (Group 1); reduced vermian area and/or BV-angle of 25°-45° (Group 2); and reduced vermian area and BV-angle > 45° (Group 3 (Dandy-Walker malformation group)).

**Table S2** Number of additional pathologic findings in study groups

| Group | Control | 1 | 2 | 3 | Total |
| --- | --- | --- | --- | --- | --- |
| No additional pathological finding | 48 | 57 | 9 | 23 | 137 |
| Renal pathology (Cyst, Agenesis, Hydronephrosis) | 27 | 2 | 1 | 1 | 31 |
| Vitium cordis | 13 | 2 | 1 | 8 | 24 |
| Congenital diaphragmatic hernia | 14 | 0 | 0 | 0 | 14 |
| Ventriculomegaly | 0 | 5 | 2 | 6 | 13 |
| Lung pathology (Sequester, CPAM) | 12 | 0 | 0 | 0 | 12 |
| (Partial) callosal agenesis | 0 | 2 | 4 | 5 | 11 |
| Gastroschisis | 8 | 0 | 0 | 0 | 8 |
| Cleft palate | 5 | 1 | 1 | 0 | 7 |
| Limb malformation (Club feet) | 5 | 0 | 2 | 0 | 7 |
| Altered gyration | 0 | 1 | 3 | 3 | 7 |
| Retrognathia | 1 | 1 | 0 | 3 | 5 |
| Sacrococcygeal teratoma | 4 | 0 | 0 | 0 | 4 |
| Meningocele | 0 | 0 | 2 | 2 | 4 |
| Germinal matrix hemorrhage | 0 | 3 | 0 | 1 | 4 |
| Subependymal heterotopia | 0 | 4 | 0 | 0 | 4 |
| Liver pathology | 3 | 0 | 0 | 0 | 3 |
| Schizencephalic cleft | 0 | 1 | 1 | 1 | 3 |
| Adrenal gland lesion | 2 | 0 | 0 | 0 | 2 |
| Mesenterial cyst | 2 | 0 | 0 | 0 | 2 |
| Ovarian cyst | 2 | 0 | 0 | 0 | 2 |
| Omphalocele | 1 | 0 | 1 | 0 | 2 |
| Rectal / Anal atresia | 1 | 1 | 0 | 0 | 2 |
| Inguinal hernia | 1 | 0 | 0 | 0 | 1 |
| Persisting right umbilical vein | 1 | 0 | 0 | 0 | 1 |
| Lymphangioma | 1 | 0 | 0 | 0 | 1 |
| Tectum malformation | 0 | 0 | 1 | 0 | 1 |
| Germinolytic cyst | 0 | 0 | 1 | 0 | 1 |
| Small configured temporal lobes | 0 | 0 | 1 | 0 | 1 |
| Abdominal situs inversus | 0 | 0 | 0 | 1 | 1 |
| Dysplastic ears | 0 | 0 | 0 | 1 | 1 |
| Periventricular cyst | 0 | 0 | 0 | 1 | 1 |

Some fetuses had more than one additional finding and are therefore represented in more than one row.
